# Directly transforming copper (I) oxide bulk into isolated single-atom copper sites catalyst through gas-transport approach

**DOI:** 10.1038/s41467-019-11796-4

**Published:** 2019-08-19

**Authors:** Zhengkun Yang, Bingxu Chen, Wenxing Chen, Yunteng Qu, Fangyao Zhou, Changming Zhao, Qian Xu, Qinghua Zhang, Xuezhi Duan, Yuen Wu

**Affiliations:** 1 School of Chemistry and Materials Science, iChEM, Hefei National Laboratory for Physical Sciences at the Microscale, University of Science and Technology of China, 230026 Hefei, China; 2 State Key Laboratory of Chemical Engineering, East China University of Science and Technology, 130 Meilong Road, 200237 Shanghai, China; 3 Beijing Key Laboratory of Construction Tailorable Advanced Functional Materials and Green Applications, School of Materials Science and Engineering, Beijing Institute of Technology, 100081 Beijing, PR China; 4 National Synchrotron Radiation Laboratory (NSRL), 230026 Hefei, China; 5 China Institute of Physics, Chinese Academy of Sciences, 100190 Beijing, China; 6 Fujian Institute of Innovation, Chinese Academy of Sciences, Beijing, China

**Keywords:** Catalyst synthesis, Electrocatalysis, Synthesis and processing

## Abstract

Single-atom metal catalysts have sparked tremendous attention, but direct transformation of cheap and easily obtainable bulk metal oxide into single atoms is still a great challenge. Here we report a facile and versatile gas-transport strategy to synthesize isolated single-atom copper sites (Cu ISAS/NC) catalyst at gram levels. Commercial copper (I) oxide powder is sublimated as mobile vapor at nearly melting temperature (1500 K) and subsequently can be trapped and reduced by the defect-rich nitrogen-doped carbon (NC), forming the isolated copper sites catalyst. Strikingly, this thermally stable Cu ISAS/NC, which is obtained above 1270 K, delivers excellent oxygen reduction performance possessing a recorded half-wave potential of 0.92 V vs RHE among other Cu-based electrocatalysts. By varying metal oxide precursors, we demonstrate the universal synthesis of different metal single atoms anchored on NC materials (M ISAS/NC, where M refers to Mo and Sn). This strategy is readily scalable and the as-prepared sintering-resistant M ISAS/NC catalysts hold great potential in high-temperature applications.

## Abstract (web summary)

Single-atom catalysts attract lots of attention, but direct transformation of bulk metal oxide into single atoms remains challenging. Here the authors report a gas-transport route to transform monolithic copper (I) oxide into copper single-atoms catalyst with a high activity for oxygen reduction.

## Introduction

Isolated single-atom sites catalysts (ISASC) have attracted a great deal of research interests, because of their superior activity and selectivity for many chemical reactions^1–3^. A variety of synthetic methods, including physical and chemical routes, have been developed to fabricate ISASC in recent years. The physical approaches, such as atomic layer deposition (ALD)^4,5^, mass-selected soft-landing technique^6^, face the drawbacks of low yields, complicated equipments and high costs, hindering their wide applications. The traditional chemical routes, such as wet impregnation^7,8^, coprecipitation, and photodeposition^9–11^, usually involve tedious synthetic steps, including adsorption and further reduction of metal precursors and stabilization on defect-rich supports. Moreover, due to the lack of strong interaction between single atoms and supports, aggregation of single atoms into clusters or nanoparticles is inevitable to some extent under a real catalytic condition, especially the high reaction temperature, thus hampering the practical industrial applications of ISASC. Therefore, to satisfy the industrial requirements such as large-scale production and excellent repeatability, developing advanced synthetic methodology is urgently required but remains challenge for the preparation of ISASC.

Recently, the direct conversion from nanoparticles to ISASC is regarded as a promising strategy since a pioneering work reported by Datye and co-workers, in which platinum single atoms anchored on CeO_2_ nanorods were constructed by thermal diffusion from platinum nanoparticles (NPs)^12^. Subsequently, Wei et al.^13^ also reported the direct transformation of noble metal NPs-to-single atom by in situ environmental transmission electron microscopy. Yang et al.^14^ described that the Ni NPs distributed on the surface of defect-containing N-doped carbon can be converted into surface-bound single Ni atoms. But studies of the direct fabrication of ISASC from cheap and readily available bulk metal materials have rarely been reported. Recently, our group demonstrated an atoms emitting and trapping strategy that can transform bulk transition metals (Cu, Co, and Ni foils) to metal ISASC with assistance of NH_3_^15^. However, metals generally exist in the form of oxide minerals under natural condition. Meanwhile, the utilization of corrosive NH_3_ will lead to harsh experiments and increase its cost to synthesize ISASC. Therefore, the direct construction of ISASC from cost-effective and commercial bulk metal oxide under non-corrosive gas protection is a more convenient route but not been achieved.

**Fig. 1:**
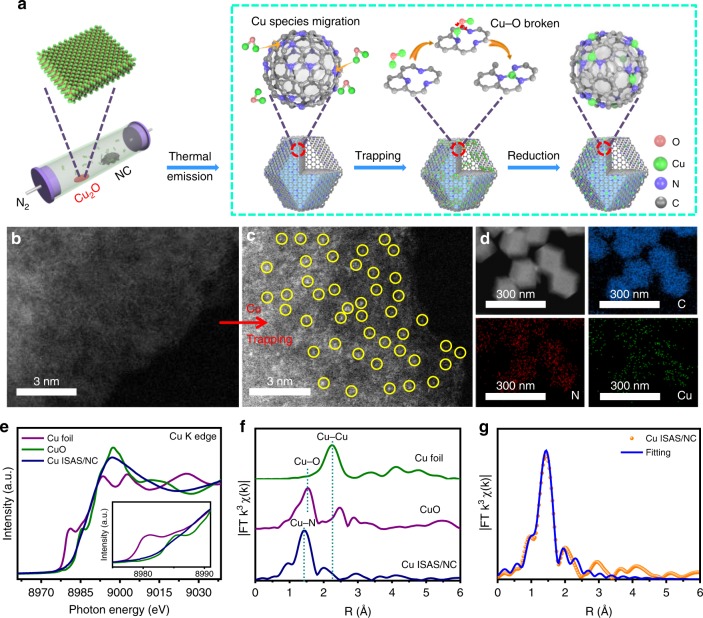
Synthesis and structural characterizations. **a** Scheme of the formation isolated copper sites (Cu ISAS/N-C) catalyst. **b** aberration-corrected high-angle annular dark-field scanning transmission electron microscope (AC HAADF-STEM) image of NC. **c** AC HAADF-STEM image of Cu ISAS/NC. **d** Corresponding EDS mapping of Cu ISAS/NC. **e** Cu K-edge X-ray absorption near-edge structure (XANES) and **f** FT k^3^-weighted extended X-ray absorption fine structure (EXAFS) spectra of Cu ISAS/NC and the reference samples. **g** Corresponding FT-EXAFS fitting curves of Cu ISAS/NC

Herein, we demonstrate a high-temperature gas-transport strategy to directly transform a series of commercial available metal oxides into isolated single atoms onto the nitrogen-doped carbon (NC) with ease of mass-production. As shown in Fig. 1a, commercial copper (I) oxide (Cu_2_O) power and NC are separately located in the porcelain boat. At 1273K in flowing N_2_, the surface Cu_2_O is initially evaporated to form volatile species, which can be trapped and reduced by the N-riched carbon support, forming the isolated Cu ISAS/NC catalyst. The volatility of Cu_2_O leads to the avoidance of corrosive NH_3_, benefiting for the large-scale production and practical applications. Importantly, a series of M ISAS/NC (M = Mo, Sn) can be fabricated by changing metal oxide precursors, demonstrating its generality to construct a variety of functional ISASC.

## Results

### Synthesis and characterization of Cu ISAS/NC

The NC matrix with abundant N-rich vacancies was prepared by a simple pyrolysis of ZIF-8 metal-organic frameworks (Supplementary Figs. 1–3) by selectively removing the volatile Zn nodes. As shown in Fig. 1b, aberration-corrected high-angle annular dark-field scanning transmission electron microscope (AC HAADF-STEM) image exhibits that there is no observable heavier metal atoms on the NC substrate. Supplementary Figure 4 shows that the Cu ISAS/NC retains the initial rhombododecahedral morphology and displays homogeneous size distribution, indicative of the trapping of Cu atoms would not result in the surface distortion and architecture collapsion. The AC HAADF-STEM images (Fig. 1c and Supplementary Fig. 5) present high-density of individual bright dots, validating the Cu species are atomically dispersed on the support. The energy-dispersive X-ray spectroscopy (EDS) analysis reveals the uniform distribution of Cu, C and N (Fig. 1d). The actual content of Cu is about 0.45% measured by inductively coupled plasma atomic emission spectroscopy (ICP-AES) analysis. X-ray diffraction (XRD) pattern of Cu ISAS/NC displays a broad peaks at 26° (Supplementary Fig. 6a), which is related to the (002) plane of graphitic carbon^16,17^. No peaks of impurities such as Cu and CuO crystals were detected, in accordance with Raman spectrum (Supplementary Fig. 6b). X-ray photoelectron spectroscopy (XPS) spectrum of Cu ISAS/NC indicates the existence of C, N and O and Cu (Supplementary Fig. 7). The high-resolution N1s (Supplementary Fig. 8a) can be deconvoluted into pyridinic-N (398.4 eV), pyrrolic-N (399.9 eV) and graphitic-N (400.8 eV)^18–20^. From the high-resolution C1s spectrum (Supplementary Fig. 8b), three peaks are ascribed to graphitic sp^2^ carbon and nitrogen-bonded carbon^21^.

### Atomic structure analysis of Cu ISAS/N-C by XAFS

To gain the chemical state and coordination environment of Cu ISAS/NC in atomic insight, X-ray absorption near-edge structure (XANES) and extended X-ray absorption fine structure (EXAFS) were conducted. The Cu K-edge XANES profiles in Fig. 1e suggest the oxidation valence state of the isolated single Cu atoms in Cu ISAS/NC is likely to be higher than metallic Cu^0^ and lower than Cu^2+^. The FT-EXAFS curve of Cu ISAS/NC sample (Fig. 1f) shows the main peak at approximately 1.5 Å, which is attributed to the scattering interaction between the Cu atoms and the first shell (Cu–N)^22^. The WT plot of Cu ISAS/NC (Supplementary Fig. 9) just displays the intensity maximum at 5 Å^−1^, assigning to the Cu-N coordination. The local atomic structure around Cu in Cu ISAS/NC by EXAFS fitting matches well with the Cu-N_3_ model (Fig. 1g, Supplementary Fig. 10 and Supplementary Table 1). The room temperature electron paramagnetic resonance (EPR) reveals the coordinatively unsaturated state of Cu species (Supplementary Fig. 11), demonstrating the existence of carbon vacancies. The N K-edge near-edge X-ray absorption fine structure (NEXAFS) spectrum shows three obvious peaks (Supplementary Fig. 12a), which result from π* transition in the C-N-C portion of the pyridinic-N site (399.5 eV) and N-3C bridging of the graphitic-N site (402.4 eV), and σ* transition of the C−N bond (408.5 eV)^23,24^. For C K-edge NEXAFS spectrum shown in Supplementary Fig. 12b, the peak A (285.6 eV) and peak B (285.6 eV) derive from π* excitations of C = C (ring) and C-N-C, respectively, and the peak C (293.2 eV) originates from C−C σ* (ring) transition^25,26^.

### Electrocatalytic ORR performance of Cu ISAS/NC

**Fig. 2:**
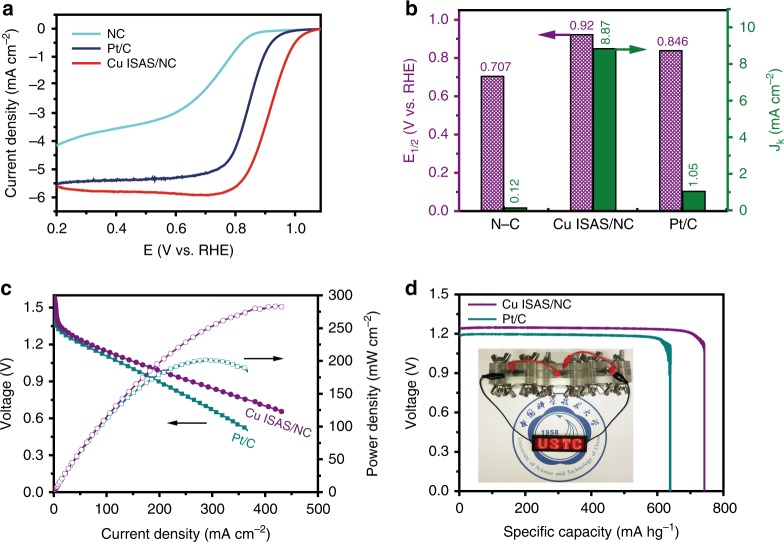
Electrocatalytic oxygen reduction and Zn-air battery performances. **a** Linear sweep voltammetry (LSV) curves of NC, Cu ISAS/NC and Pt/C catalysts in 0.1 M KOH solution with a sweep rate of 10 mV s^−1^ and rotation rate of 1600 rpm. **b** half-wave potential (*E*_1/2_) and kinetic current density (*J*_K_) of different catalysts. **c** Polarization and corresponding power density plots of Cu ISAS/NC and Pt/C-based Zn-air batteries. **d** The specific capacity of Cu ISAS/NC and Pt/C-based Zn-air batteries at 50 mA cm^−2^. (Inset: a photograph showing light-emitting diode panel powered by three Cu ISAS/NC-based Zn–air batteries)

Nitrogen (N_2_) adsorption/desorption isotherms demonstrate that Cu ISAS/NC has a high Brunauer−Emmett−Teller (BET) surface area of 831 m^2^ g^−1^ (Supplementary Fig. 13), due to their highly porous structure. Thus, the Cu ISAS/NC catalyst offers high accessible surface area and large exposed active sites, facilitating the mass transport. According to the BET and ICP-AES analysis, the surface coverage of Cu atoms is estimated to be 0.0509 atoms/nm^2^. The oxygen reduction reaction (ORR) performance of NC, Cu ISAS/NC and commercial Pt/C was evaluated by linear sweep voltammetry (LSV) technology in an O_2_-saturated 0.1 M KOH condition. As displayed in Fig. 2a, after Cu atoms doping, the Cu ISAS/NC catalyst provides the higher ORR activity with half-wave potential (E_1/2_) of (0.92 V vs RHE) (Fig. 2b), which is 52 mV higher than that of Pt/C. This value is among the best ORR activity delivered by noble metal-free catalysts reported previously (Supplementary Table 2). At a potential of 0.9 V, Cu ISAS/NC catalyst exhibits a much larger kinetic current density (8.87 mA cm^−2^) than those of Pt/C (1.05 mA cm^−2^) and NC (0.12 mA cm^−2^). Additionally, the highest ORR kinetic process catalyzed by Cu ISAS/NC was further evidenced by a smaller Tafel slope of 59 mV dec^−1^ compared with that of Pt/C (95 mV dec^−1^) and NC matrix (116 mV dec^−1^) (Supplementary Fig. 14). The value of electron transfer number (*n*) was calculated to be 3.99 (Supplementary Fig. 15), close to the theoretical value of 4.0 for oxygen reduction. The rotating ring-disk electrode (RRDE) tests further confirmed the four-electron pathway of Cu ISAS/NC catalyst in KOH (Supplementary Fig. 16). Additionally, the Cu ISAS/NC displays excellent stability after 20,000 cycles (Supplementary Fig. 17). This excellent robustness is ascribed to the highly stable atomic reactive sites, whose atomic dispersion remain after the durability (Supplementary Fig. 18). The Cu ISAS/NC could easily be scaled up to higher yields to satisfy the demand of large-scale production (Supplementary Fig. 19). To validate the implementation, the Cu ISAS/NC material was employed into a primary Zn–air battery (Supplementary Fig. 20). As exhibited in Fig. 2c, the Zn–air battery using Cu ISAS/NC catalyst displays high performance with the maximum power density of up to 280 mW cm^−2^, superior to Pt/C-based Zn-air battery (200 mW cm^−2^), as well as other reported catalysts (Supplementary Table 3). At the discharge of 50 mA cm^−2^ (Fig. 2d), the specific capacity of the Zn-air battery using the Cu ISAS/NC as air-cathode was estimated to be ~736 mAh g^−1^. More importantly, the Cu ISAS/NC-based battery can robustly serve over 45 h with only a negligible drop of discharge voltage (Supplementary Fig. 21), indicating the excellent stability of the Cu ISAS/NC catalyst in practical Zn-air device.

### DFT calculations

**Fig. 3:**
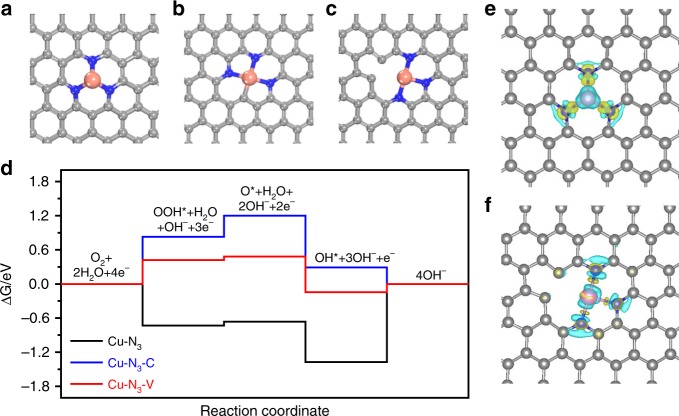
DFT calculation. Top view of the optimized structures of the models of **a** Cu-N_3_, **b** Cu-N_3_-C and **c** Cu-N_3_-V. Gray, blue and orange represent C, N, and Cu atoms, respectively. **d** Free energy diagram for ORR process on these three models at the equilibrium potential (*U* = 0.40 V vs. NHE or *U* = 1.23 V vs RHE) at pH = 14. The corresponding charge density difference of **e** Cu-N_3_ and **f** Cu-N_3_-V, in which light blue and yellow isosurfaces denote a decrease and increase of 0.005 e/Å^3^ for electronic density, respectively

We further executed density functional theory (DFT) calculations to explore the nature of the active sites of the Cu ISAS/NC ORR catalyst. Based on the EXAFS analysis for the Cu ISAS/NC, we proposed two models containing Cu-N_3_ structure, as shown in Fig. 3a, b, where the Cu in the Cu ISAS/NC is coordinated by three N atoms (Cu-N_3_) as well as one C atom and three N atoms (Cu-N_3_-C), respectively. Considering that the carbon defect may be induced during the reduction of Cu-O species, a Cu-N_3_ model with a vacancy (Cu-N_3_-V) was also constructed (Fig. 3c). According to the associative mechanism in alkaline medium and the correspondingly optimized configurations of the intermediates (Supplementary Fig. 22–24) as well as the free energies for each step (Supplementary Table 4) for the ORR, the free energy diagrams of ORR processes were subsequently obtained at the equilibrium potential (U = 0.40 V vs NHE at the pH = 14) over the single Cu active sites of these models and the results are shown in Fig. 3d. Clearly, for the Cu-N_3_ model, the OH* removal is the rate-determining step, while for the Cu-N_3_-C and Cu-N_3_-V models, the OOH* formation is the rate-determining step. Furthermore, the theoretical ORR overpotentials of these three models, an important measure of the ORR catalyst performance^27^, were obtained from the correlated free energy profiles (Supplementary Fig. 25). Obviously, both the Cu-N_3_ and the Cu-N_3_-C models possess high theoretical ORR overpotentials (1.37 eV and 0.83 eV), which are much higher than the overpotential of the Cu ISAS/NC catalyst in our experiment. Interestingly, the theoretical overpotential of the Cu-N_3_-V model, which is derived from introducing defect in Cu-N_3_-C model, decreased darmatically compared to that of the Cu-N_3_ and Cu-N_3_-C models, indicating the important role of defect in the Cu-N-C ORR catalysts. Notably, even the defect is trapped by the adsorbed oxygen (O*) during the ORR process (Supplementary Fig. 26), the Cu-N_3_-V model with O* pre-adsorbed on the vacancy still show relatively low theoretical overpotential (0.517V), indicating the Cu-N3-V model as the possible active site of the Cu ISAS/NC ORR catalyst. The charge density difference shown in Fig. 3e, f indicate that the introduction of the defect will cause the inhomogeneous charge distribution around the Cu SAs. Such charge density asymmetry will lead to the synergistic effect of the defect and the N coordination around the Cu SAs and thus enhance the ORR activity^19,28^.

**Fig. 4:**
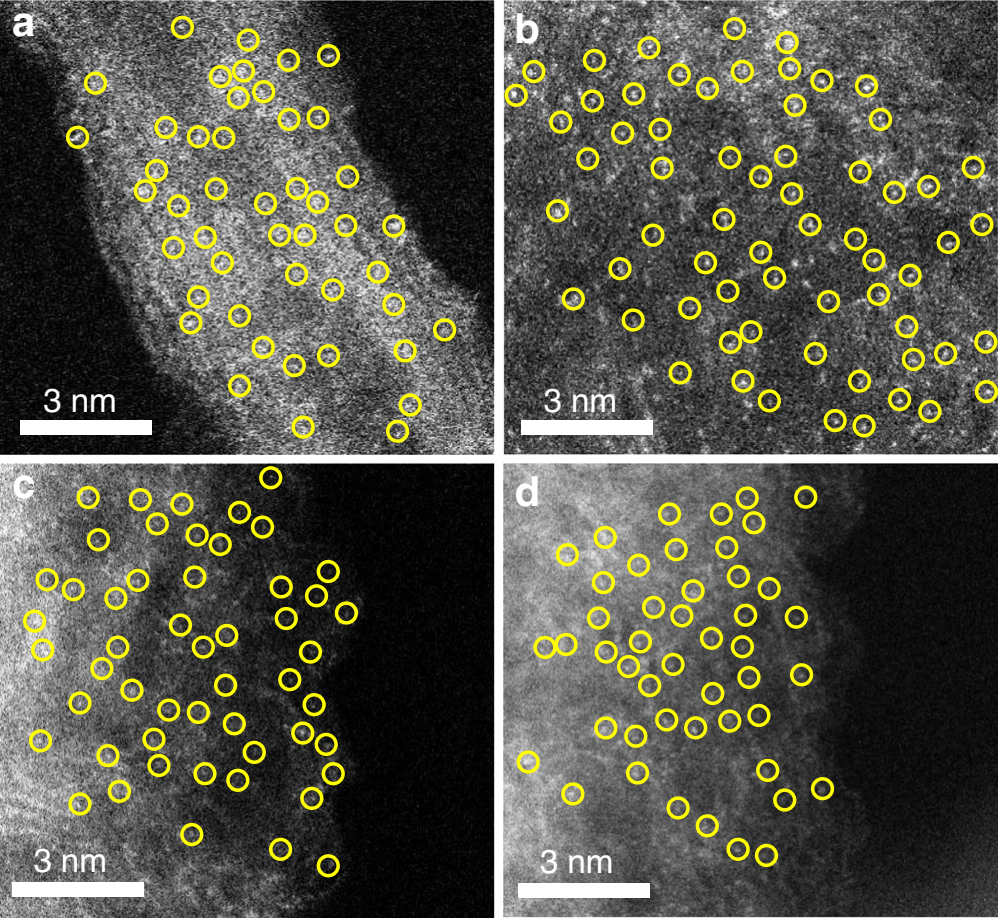
Atomic structural characterizations AC HAADF-STEM images of **a** Cu ISAS/N-CNTs, **b** Cu ISAS/N-rGO, **c** Mo ISAS/NC, and **d** Sn ISAS/NC catalysts

Interestingly, when the NC trapping agent was substituted by N-doped reduced graphene oxide (N-rGO) and N-doped carbon nanotubes (N-CNTs), we also successfully obtained isolated Cu ISAS/N-rGO (Fig. 4a, Supplementary Fig. 27 and Fig. 28) and Cu ISAS/N-CNTs (Fig. 4b, Supplementary Fig. 29 and Fig. 30) catalysts. The ORR performance for the Cu ISAS/N-CNTs and Cu ISAS/N-rGO are shown in Fig. S31. To test the universality of the high-temperature gas-transport route, we used different metal oxide powders (metal = Mo, Sn) to fabricate isolated M ISAS/NC materials. The AC HAADF-STEM images distinctly present isolated bright dots in Mo ISAS/NC (Fig. 4c and Supplementary Fig. 32) and Sn ISAS/NC (Fig. 4d and Supplementary Fig. 33), validating the atomically dispersed Mo and Sn atoms anchored on N-doped carbons.

## Discussion

We have demonstrated a versatile and universal gas-transport route to directly transform monolithic metal oxides into isolated single-atoms electrocatalysts. Under high temperature, the surface metal oxides were initially evaporated to generate volatile species, which were trapped and reduced by the N-rich carbon supports, forming the isolated M ISAS/NC catalysts (M = Cu, Mo, Sn). Impressively, the as-prepared Cu ISAS/NC catalyst has been demonstrated to endow excellent catalytic activity for ORR in an alkaline medium and achieve high performance for a Zn–air battery. This work paves a way to directly prepare single atoms from bulk metal oxides.

## Methods

### Chemicals

Copper oxide (Cu_2_O), Molybdenum trioxide (MoO_3_), tin dioxide (SnO_2_) melamine (M) were obtained from Sinopharm Chemical Reagents, China. Multi-walled carbon nanotubes (CNTs) was purchased from Alfa Aesar. Graphite powder (400 mesh) was obtained from XFNANO. Toray Carbon Paper (Toray TGP-H^−^060, Toray Industries Inc.) was ultrasonically cleaned in ethanol. Analytical grade methanol (CH3OH), ethanol, zinc nitrate hexahydrate (Zn(NO_3_)_2_.6H_2_O), 2-methylimidazole were purchased from Aldrich. All the chemicals were analytical grade and used without further purification.

### Synthesis of NC

In a typical procedure, 3 g Zn(NO_3_)_2_•6H_2_O was dissolved in 50 ml methanol and subsequently added into 100 ml methanol containing 6.5 g 2-methylimidazole (MeIM) under vigorous stirring for 24 h at room temperature. The as-obtained precipitates were centrifuged and washed with methanol three times and dried in vacuum at 60 °C for overnight. The dried sample was placed in the porcelain boat. Then, the boat was heated at 950 °C under N_2_ for 1 h with the heating rate of 5 °C/min. After the temperature was down to room temperature, the mixture was stored in the glass bottle for further use.

### Synthesis of Cu ISAS/NC

In a normal procedure, the Cu_2_O power and the powder of NC (100 mg) were separately placed on the porcelain boat. The porcelain boat was placed in a tube furnace and heated to 1000 °C (heating rate 5 °C/min) in a stream of N_2_ (10 ml/min) for 5 h to yield Cu ISAS/NC.

### Synthesis of Cu ISAS/N-CNTs and Cu ISAS/N-rGO

The nitrogen-doped CNTs (N-CNTs) and nitrogen-doped reduced graphene oxide (N-rGO) were prepared by pyrolysis the mixtures of CNTs and M (mass ration: 1:2) and GO and M (mass ration: 1:2), respectively, at 1000 °C for 1 h in flowing N_2_. Synthesis of Cu ISAS/N-CNTs and Cu ISAS/N-rGO were the same as Cu ISAS/NC except using N-CNTs or N-rGO as support.

### Synthesis of Mo SAs/NC and Sn SAs/NC

The preparation of Mo ISAS/NC and Sn ISAS/NC is similar as Cu ISAS/NC except using MoO_3_ and SnO_2_ power. The temperature was changed as 600 °C for Mo ISAS/NC.

### Electrochemical measurements

All electrochemical tests were performed on CHI 760E electrochemical workstation with a three-electrode electrochemical cell, in which a graphite rod used as counter electrode, an Ag/AgCl as reference electrode. The catalyst ink was prepared by ultrasonically 5 mg Cu ISAS/NC catalyst powder dispersed into 1 mL ethanol including 10 μL 5% Nafion (D520, Dupont Inc., USA). A volume of 10 μL of the homogeneous catalyst ink was droped on a glassy carbon (GC) electrode with a diameter of 5 cm. A flow of O_2_ was kept over the electrolyte during the tests so as to guarantee O_2_ saturation. LSV curves of the catalysts were conducted using a rotating disk electrode (RDE) with a scan rate of 10 mV s^−1^ at a rotating speed of 1600 rpm. The overall number of electrons transferred (n) during in oxygen reduction was calculated by Koutecky-Levich equation:

1 $$\frac{1}{J} = \frac{1}{{J_{\mathrm{L}}}} + \frac{1}{{J_{\mathrm{K}}}} = \frac{1}{{B\omega ^{1/2}}} + \frac{1}{{J_{\mathrm{K}}}}$$

where J is the measured current density, *J*_K_ and *J*_L_ are the kinetic and diffusion-limiting current densities, *ω* is the angular velocity (*ω* = 2πN, *N* is the rotation speed), *B* is Levich slope which is calculated as below:

2 $${\mathrm{B}} = 0.62nFC_0D_0^{2/3}V^{ - 1/6}$$

where F is the Faraday constant (96485 C mol^−1^), *C*_0_ is the bulk concentration of O_2_ ((1.2 × 10^−6^ mol cm^−3^), *D*_0_ is the diffusion coefficient of O_2_ in 0.1 M KOH (1.9 × 10^−5^ cm^2^ s^−1^), and *ν* is the kinematic viscosity of the electrolyte (0.01 cm^2^ s^−1^). Rotating ring-disk electrode (RRDE) measurements of the samples was measured to study the four-electron selectivity. The Pt ring electrode was biased at 1.2 V vs. RHE. The H_2_O_2_ yield and n per oxygen molecule were calculated by the following equations:

3 $${\mathrm{\% H}}_2{\mathrm{O}}_2 = 200\frac{{I_{\mathrm{R}}/N}}{{I_{\mathrm{D}} + I_{\mathrm{R}}/N}}$$

4 $$n = 4\frac{{I_{\mathrm{D}}}}{{I_{\mathrm{D}} + I_{\mathrm{R}}/N}}$$

where *I*_D_ and *I*_R_ are the disk and ring currents, respectively. *N* is the ring current collection efficiency (37%).

### Zinc-air (Zn-air) battery measurements

Primary Zn-air batteries tests were carried out in a home-built device. The air electrode was prepared by uniformly brushing the as-prepared catalyst ink onto carbon fiber paper (1.0 cm^2^) at 60 °C. All the catalyst loading of the air cathode with the Cu ISAS/NC and Pt/C catalysts was 1.0 mg cm^−2^ unless otherwise stated. The polished commercial Zn foil was used as anode. The electrolyte in home-built Zn–air battery is 6 M KOH electrolyte saturated with O_2_. The discharge polarization curve and stabilitly measurements were measured using the as-made device with CHI 760E electrochemical workstation.

### Computational details

All DFT calculations were conducted employing the Vienna ab initio simulation package (VASP)^29–32^. We used the projector augmented wave (PAW) method to depict the interactions between ion cores and valence electrons^33^. Meanwhile, we used the GGA-PBE to describe the exchange-correlation functional^34,35^. The solution of the Kohn-Sham equations was expanded in a plane wave basis set with a cutoff energy of 400 eV. The Brillouin zone sampling was performed using a Monkhorst-Pack grid^36^, and electronic occupancies were determined in light of a Gaussian smearing with a width of 0.05 eV. Considering that the solvent may influence the calculation results, we employed VASPsol to include the implicit solvation effect in the calculations^37^. A force-based conjugated gradient method was used to optimize the geometries in all the calculations^38^. Saddle points and minima were converged when the maximum force in each degree of freedom was less than 0.03 eV Å^−1^. Bader charge analysis was implemented with a fast algorithm developed by Henkelman and coworkers, and the core charges were included in the partitions^39,40^.

All of the Cu ISAS supported on the graphene models are constructed based on the graphene basal plane model with the supercell of 5 × 3$\sqrt 3$× 1 (12.30 Å × 12.78 Å × 15.00 Å). The Monkhorst-Pack meshes of 3 × 3 × 1 k-point samplings in the surface Brillouin zones were used for these models. All models constructed are shown in Supplementary Fig. 21. The ORR were further analyzed on the Cu sites of these models. The ORR can proceed through a two-electron pathway in which O_2_ is reduced to H_2_O_2_, or a four-electron process that completely reduces O_2_ to H_2_O. Here, we calculate the complete reduction cycle based on the experimental observations that the four-electron was the dominant mechanism on the Cu ISAS/NC catalyst. In alkaline condition, the ORR step can be summarized as follows:

5 $${\mathrm{O}}_2^ \ast + 2{\mathrm{H}}_{2}{\mathrm{O}} + 4e^ - \to {\mathrm{OOH}}^ \ast + {\mathrm{OH}}^ - {\mathrm{H}}_{2}{\mathrm{O}} + 3e^ -$$

6 $${\mathrm{OOH}}^ \ast + {\mathrm{OH}}^ - + {\mathrm{H}}_{2}{\mathrm{O}} + 3e^ - \to 2{\mathrm{OH}}^ - + {\mathrm{O}}^ \ast + {\mathrm{H}}_{2}{\mathrm{O}} + 2e^ -$$

7 $$2{\mathrm{OH}}^ - + {\mathrm{O}}^ \ast + {\mathrm{H}}_{2}{\mathrm{O}} + {\mathrm{2}}e^ - \to 3{\mathrm{OH}}^ - + {\mathrm{OH}}^ \ast + e^ -$$

8 $$3{\mathrm{OH}}^ - + {\mathrm{OH}}^ \ast + e^ - \to 4{\mathrm{OH}}^ -$$

9 $${\mathrm{Overall}}:{\mathrm{O}}_2 + 2{\mathrm{H}}_{2}{\mathrm{O}} + 4e^ - \to 4{\mathrm{OH}}^ -$$

In ORR, the rate-determining steps were reported as the adsorption of O_2_ as OOH* (5) or the desorption of OH* (7)^41^ and at all the steps would influence the corresponding ORR activity. Here, we used these reactions to derive the ORR thermochemistry. The free energy diagrams for ORR were determined according to the method proposed by Nørskov et al.^27^. We drew the free energy diagrams by setting up the reference electrode as the NHE. It is noted that the equilibrium potential U^0^ for ORR at pH = 14 was determined to be 0.402 V vs NHE, where the reactant and product are at the same energy level^42^. In our calculations, the ORR was analyzed using intermediate species associated with one electron transfer at a time. The free energy change from initial states to final states of the reaction is calculated as follows:

10 $$\Delta {\mathrm{G}} = \Delta E + \Delta {\mathrm{ZPE}} - T\Delta S + \Delta G_{\mathrm{U}} + \Delta G_{{\mathrm{pH}}}$$

where ΔE is the reaction energy of reactants and products adsorbed on catalyst surface, ΔZPE is the change of zero-point energy, Δ*G*_U_ = −eU (*U* is the electrode potential and *e* is the transferred charge), *T* is the temperature, Δ*S* is the entropy change at 298.15 K, Δ*G*_pH_ is the correction of the H^+^ free energy, which is calculated as Δ*G*_pH_ = k_B_T × ln10 pH, where *k*_B_ is the Boltzmann constant. The free energy of H_2_, H_2_O, and O_2_ was the same as previous literature reported^27^. The free energy of (H^+^ + e^−^) in solution at standard conditions of *U* = 0 and pH = 0 is equal to that of 1/2 H_2_ according to a computational hydrogen electrode model suggested by Nørskov et al.^27^. The free energy of OH^−^ was calculated from the reaction H^+^ + OH^−^→H_2_O, which is in equilibrium in water solution^43,44^. The entropies and vibrational frequencies of the species in gas phase were taken from the previous literatures^27^. Zero-point energy and entropies of the adsorbed species were calculated from the vibrational frequencies. To acquire the reaction free energy of ORR process, we firstly calculated the adsorption free energy of O*, OH*, and OOH*. Due to the difficulies in obtaining the exact free energy of OOH, O, and OH radicals in the electrolyte solution, the adsorption free energy of these intermediates are all calculated based on the relative free energy of stoichiometrically appropriate amounts of H_2_O (g) and H_2_(g) according to the previous studies^27^. The corresponding reaction free energies of equations 5 to 8 (ΔG_1_ to ΔG_4_) are obtained based on the adsorption free energy of OOH, O, and OH and the results are shown in Supplementary Table 4. The overpotential *η*_ORR_ of the whole ORR process can be calculated at the equilibrium potential (*U* = 0.40 V vs. NHE at pH = 14) by the following equations:

11 $$\eta _{{\mathrm{ORR}}} = {\mathrm{max}}\left\{ {\Delta G_1;\Delta G_2;\Delta G_3;\Delta G_4} \right\}/e$$

## Supplementary information

Supplementary Information

Description of Additional Supplementary Files

Supplementary Dataset 1

Supplementary Dataset 2

Supplementary Dataset 3

Supplementary Dataset 4

## Data Availability

The data that support the findings of this study are available from the corresponding author upon request.
