# Supplementary material for: Directly transforming copper (I) oxide bulk into isolated single-atom copper sites catalyst through gas-transport approach
Source: Nat Commun. 2019 Aug 19;10:3734. doi: 10.1038/s41467-019-11796-4 (PMC6700197; doi:10.1038/s41467-019-11796-4)
Supplement: Supplementary file 1 — Supplementary Information [file 41467_2019_11796_MOESM1_ESM.pdf]

Directly Transforming Copper (I) Oxide Bulk into Isolated Single-Atom Copper Sites Catalyst through Gas-Transport Approach

Yang et al.

Supplementary Methods

Chemicals. Copper oxide (Cu_2O), Molybdenum trioxide (MoO_3), tin dioxide (SnO_2) melamine (M) were obtained from Sinopharm Chemical Reagents, China. Multi-walled carbon nanotubes (CNTs) was purchased from Alfa Aesar. Graphite powder (400 mesh) was obtained from XFNANO. Toray Carbon Paper (Toray TGP-H-060, Toray Industries Inc.) was ultrasonically cleaned in ethanol. Analytical grade methanol (CH_3OH), ethanol, zinc nitrate hexahydrate ($Zn(NO_3)_2.6H_2O$), 2-methylimidazole were purchased from Aldrich. All the chemicals were analytical grade and used without further purification.

Synthesis of NC. In a typical procedure, 3 g $Zn(NO_3)_2•6H_2O$ was dissolved in 50 ml methanol and subsequently added into 100 ml methanol containing 6.5 g 2-methylimidazole (MeIM) under vigorous stirring for 24 h at room temperature. The as-obtained precipitates were centrifuged and washed with methanol three times and dried in vacuum at 60 ℃ for overnight. The dried sample was placed in the porcelain boat. Then, the boat was heated at 950 ℃ under N_2 for 1 h with the heating rate of 5 ℃ /min. After the temperature was down to room temperature, the mixture was stored in the glass bottle for further use.

Synthesis of Cu ISAS/NC. In a normal procedure, the Cu_2O power and the powder of NC (100 mg) were separately placed on the porcelain boat. The porcelain boat was placed in a tube furnace and heated to 1000 ℃ (heating rate 5 ℃/min) in a stream of N_2 (10 ml/min) for 5 h to yield Cu ISAS/NC.

Synthesis of Cu ISAS/N-CNTs and Cu ISAS/N-rGO. The nitrogen-doped CNTs (N-CNTs) and nitrogen-doped reduced graphene oxide (N-rGO) were prepared by pyrolysis the mixtures of CNTs and M (mass ration: 1:2) and GO and M (mass ration: 1:2), respectively, at 1000 ℃ for 1h in flowing N_2. Synthesis of Cu ISAS/N-CNTs and Cu ISAS/N-rGO were the same as Cu ISAS/NC except using N-CNTs or N-rGO as support.

Synthesis of Mo SAs/NC and Sn SAs/NC. The preparation of Mo ISAS/NC and Sn ISAS/NC is similar as Cu ISAS/NC except using MoO_3 and SnO_2 power. The temperature was changed as 600 ℃ for Mo ISAS/NC.

Catalyst characterization. Powder X-ray diffraction patterns of samples were recorded on a Rigaku Miniflex-600 operating at 40 KV voltage and 15 mA current with Cu Kα radiation (λ=0.15406 nm). TEM was carried out by a Hitachi-7700 working at 100 kV. The high-resolution TEM, HAADF-STEM and EDS mapping were recorded by a Titan ETEM microscope (FEI) with a spherical aberration corrector working at 200 kV. The SEM was performed on JSM-6700F. XPS was collected on scanning X-ray microprobe (PHI 5000 Verasa, ULAC-PHI, Inc.) using Al Ka radiation and the C1s peak at 284.8 eV as internal standard. The electron paramagnetic resonance (EPR) spectra were recorded on a JEOL JES-FA200 EPR spectrometer. The ICP measurement was performed on Optima 7300 DV. The BET (Brunauer-Emmett-Teller) test was obtained from micromeritics ASAP 2020. The pore size distribution was calculated from the BJH method for micropore and mesoporous. Near Edge X-ray Absorption Fine Structure (NEXAFS) were carried out

at the Catalysis and Surface Science Endstation at the BL11U beamline and Photoemission endstation at the BL10B beamline in the National Synchrotron Radiation Laboratory (NSRL) in Hefei, China. The X-ray absorption find structure data (Cu K-edge) were collected at 1W1B station in Beijing Synchrotron Radiation Facility (BSRF). The storage rings of BSRF were operated at 2.5 GeV with a maximum current of 250 mA. The data were collected at room temperature in transmission mode using N_2-filled ionization chamber. All samples were pelletized as disks of 13 mm diameter using graphite powder as a binder. The acquired EXAFS data were processed according to the standard procedures using the ATHENA module implemented in the IFEFFIT software packages. The EXAFS spectra were obtained by subtracting the post-edge background from the overall absorption and then normalizing with respect to the edge-jump step. Subsequently, $\chi(k)$ data in the k-space were Fourier transformed to real (R) space using a hanning windows (dk=1.0 $Å^{-1}$) to separate the EXAFS contributions from different coordination shells.

Supplementary Figures

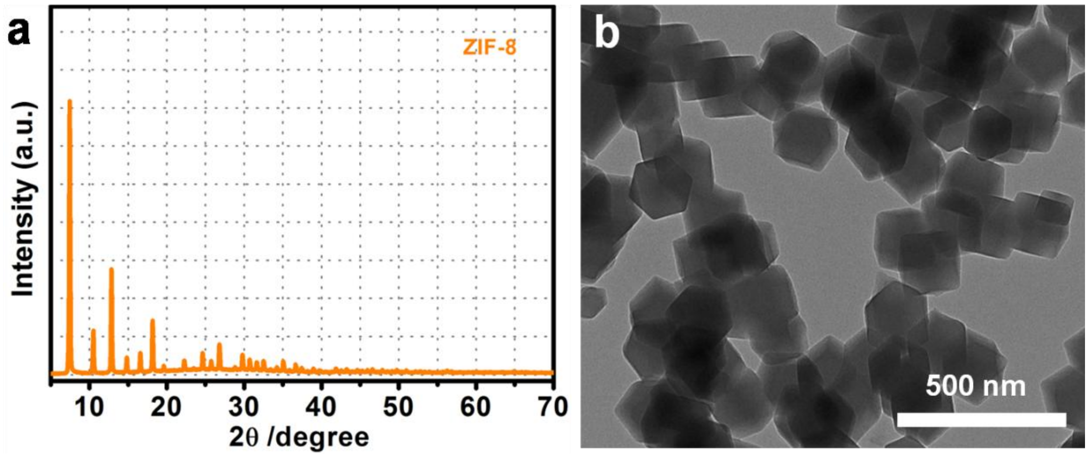

Supplementary Figure 1. The XRD pattern (a) and TEM image of ZIF-8 sample.

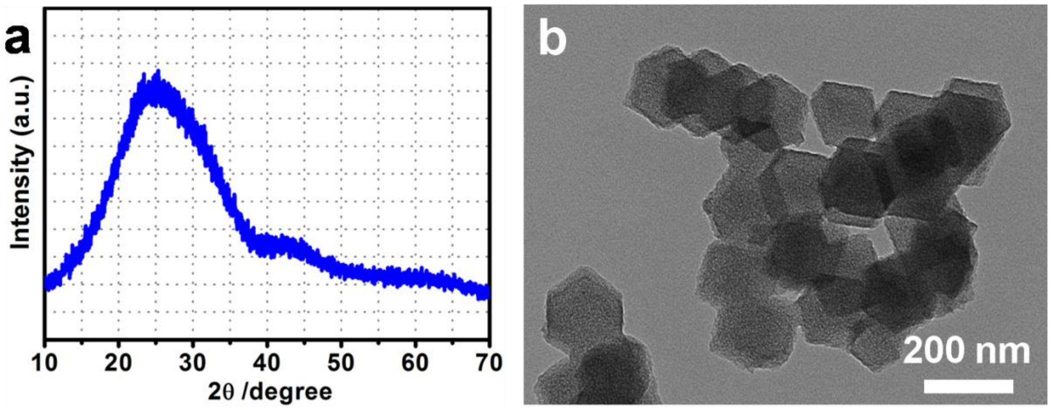

Supplementary Figure 2. The XRD pattern a) and b) TEM image of NC sample.

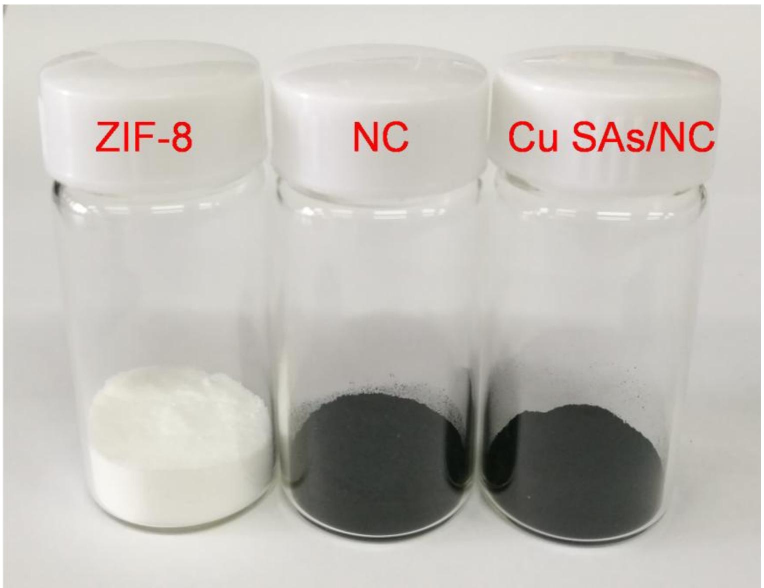

Supplementary Figure 3. The color of the ZIF-8, NC, and Cu ISAS/NC sample.

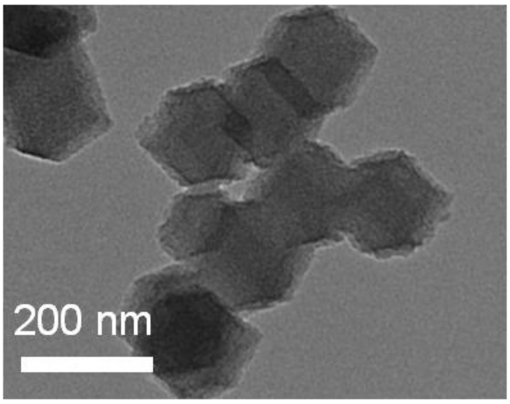

Supplementary Figure 4. The TEM image of Cu ISAS/NC catalyst.

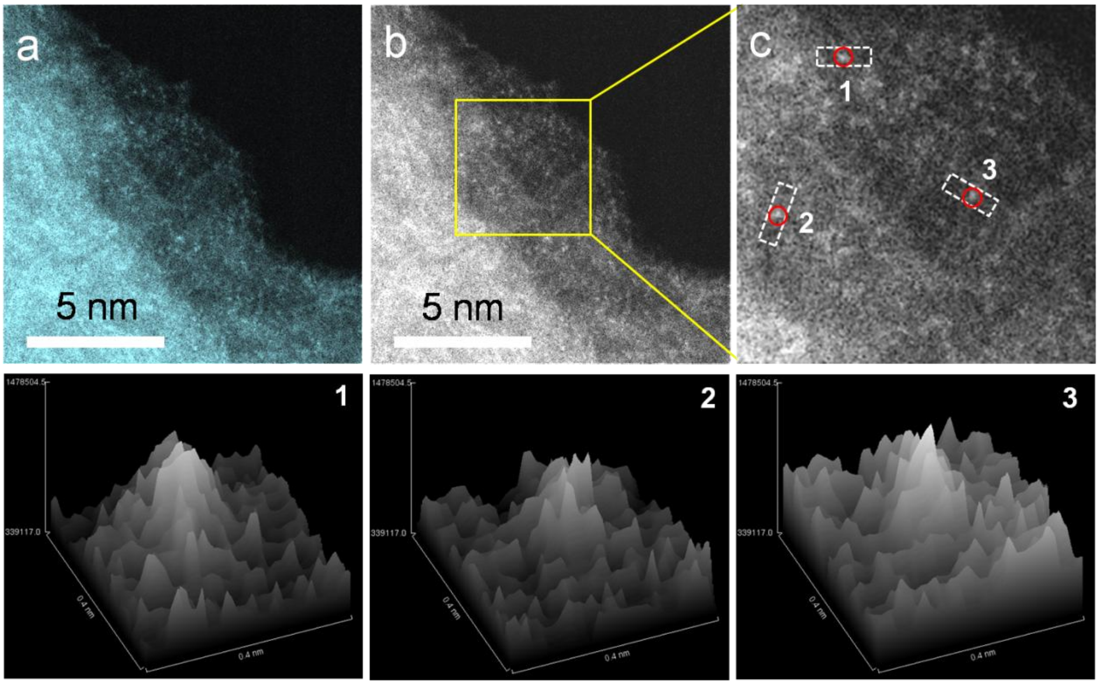

Supplementary Figure 5. a-b) AC HAADF STEM image of Cu ISAS/N-C. c) Several single atoms have been highlighted and the corresponding intensity profiles.

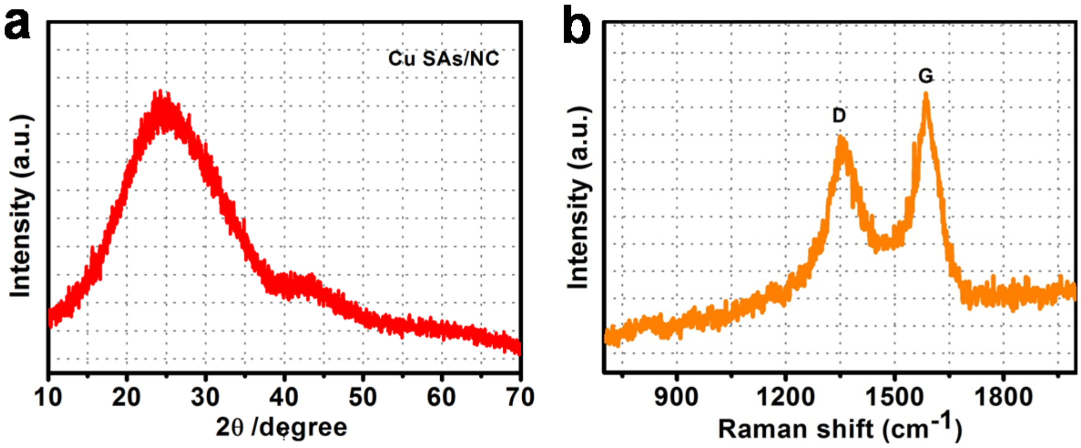

Supplementary Figure 6. a) The XRD pattern and b) Raman spectrum of Cu ISAS/NC sample.

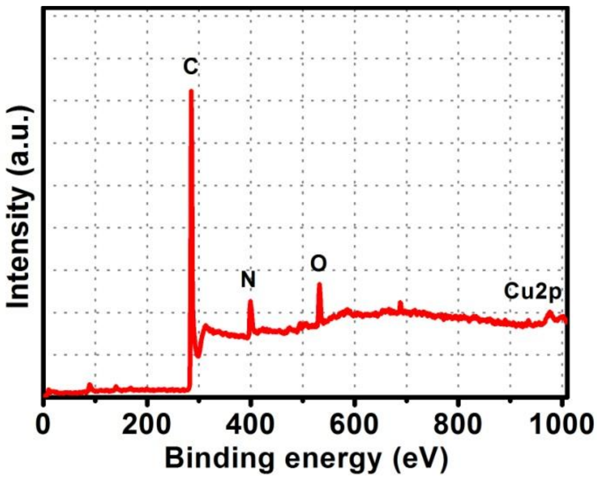

Supplementary Figure 7. The XPS survey scan of the Cu ISAS/NC sample.

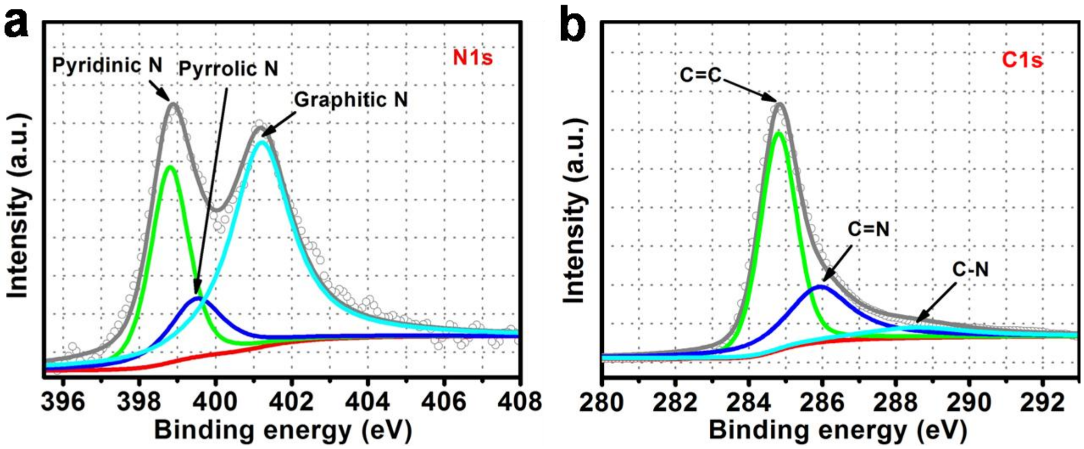

Supplementary Figure 8. XPS spectra for the a) N1s and b) C1s of Cu ISAS/NC sample.

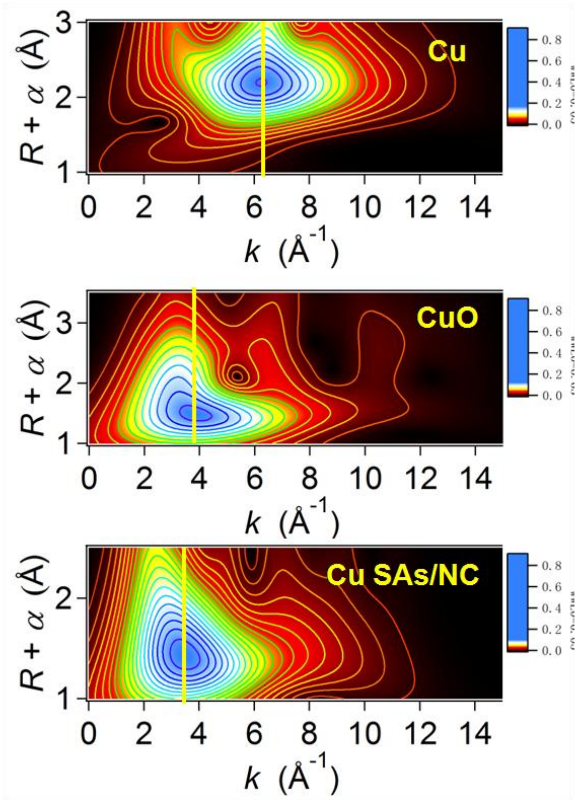

Supplementary Figure 9. WT-EXAFS of Cu foil, CuO and the Cu ISAS/NC.

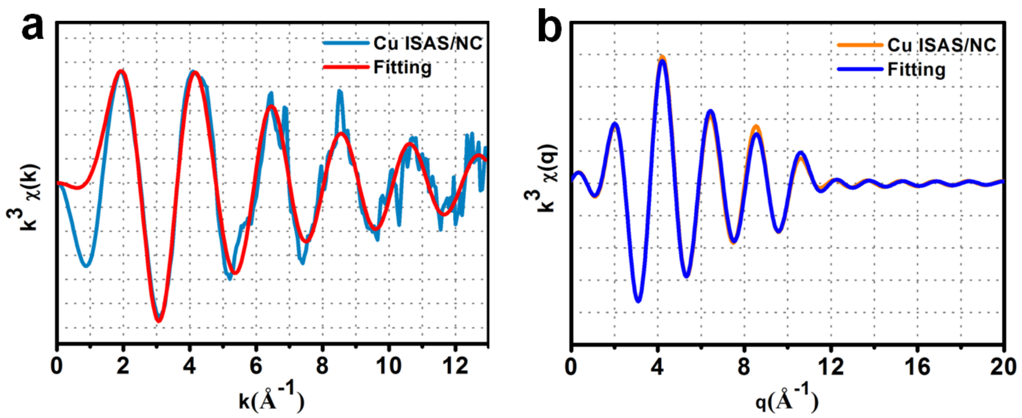

Supplementary Figure 10. The corresponding EXAFS k space a) and q space b) fitting curves of Cu ISAS/NC.

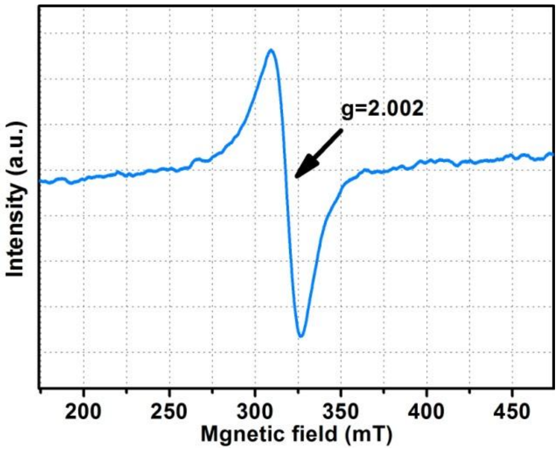

Supplementary Figure 11. EPR spectrum of Cu ISAS/NC measured at room temperature.

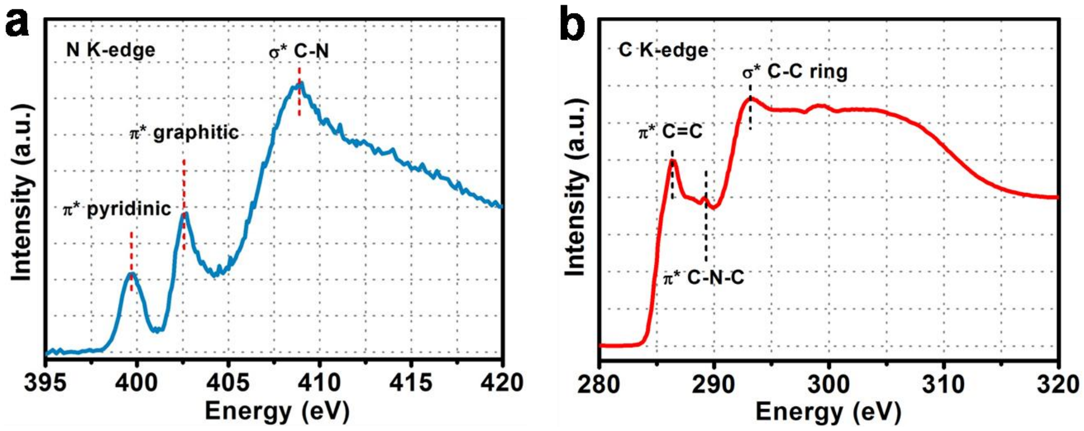

Supplementary Figure 12. a) N K-edge and b) C K-edge NEXAFS spectra of Cu ISAS/NC.

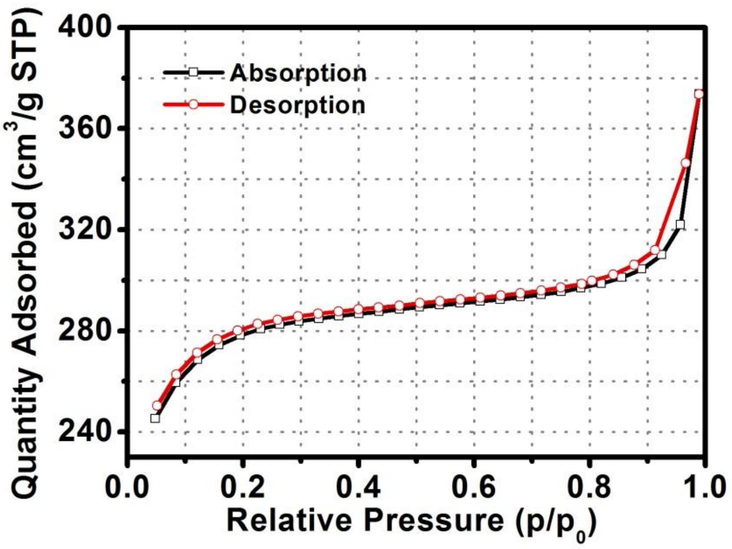

Supplementary Figure 13. N_2 adsorption-desorption isotherm curve of the Cu ISAS/NC catalyst.

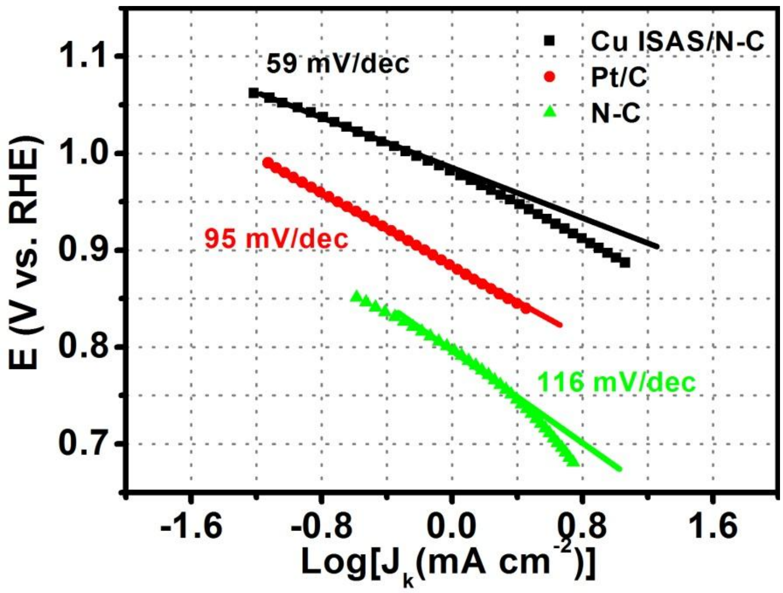

Supplementary Figure 14. Corresponding electrochemical Tafel plots for NC, Cu ISAS/NC and Pt/C obtained from the LSV curves.

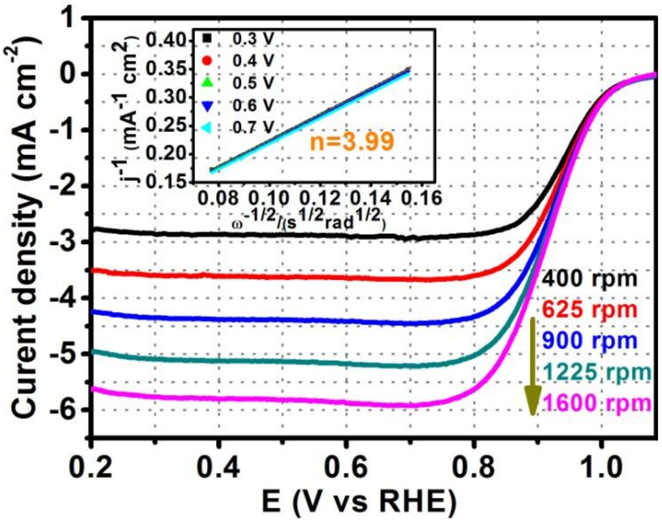

Supplementary Figure 15. LSV curves of Cu ISAS/NC at different rotation rates. Inset: K-L plots.

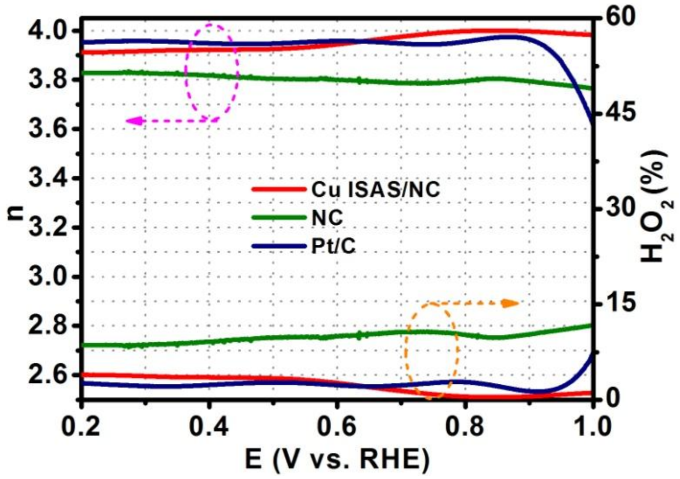

Supplementary Figure 16. H_2O_2 yield and electron transfer number (n) of different catalysts.

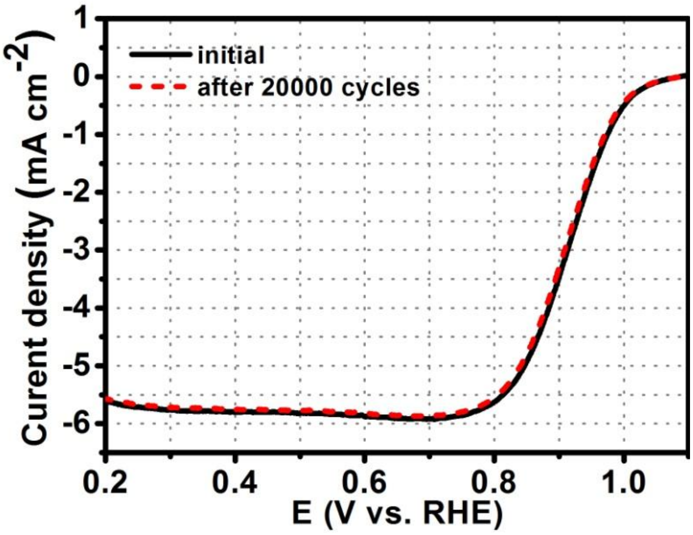

Supplementary Figure 17. Long-term stability test for Cu ISAS/NC.

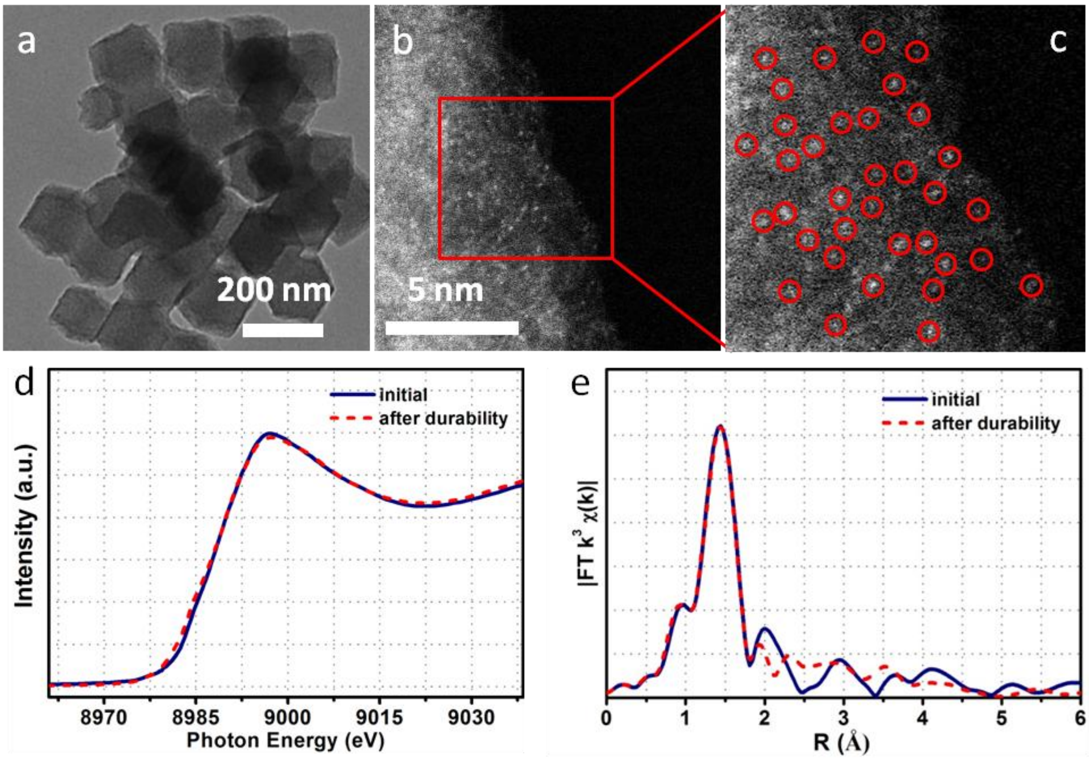

Supplementary Figure 18. a) TEM, b) Magnified HAADF STEM images of Cu ISAS/NC catalyst after durability test. The result reveals the atomically dispersed Cu atoms remained unchanged. d) Cu K-edge XANES and (e) FT k^3-weighted EXAFS spectra of Cu ISAS/NC befeor and after durability test.

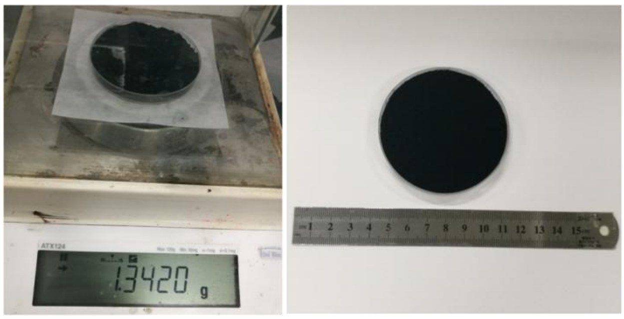

Supplementary Figure 19. Picture of higher yield of Cu ISAS/NC catalyst.

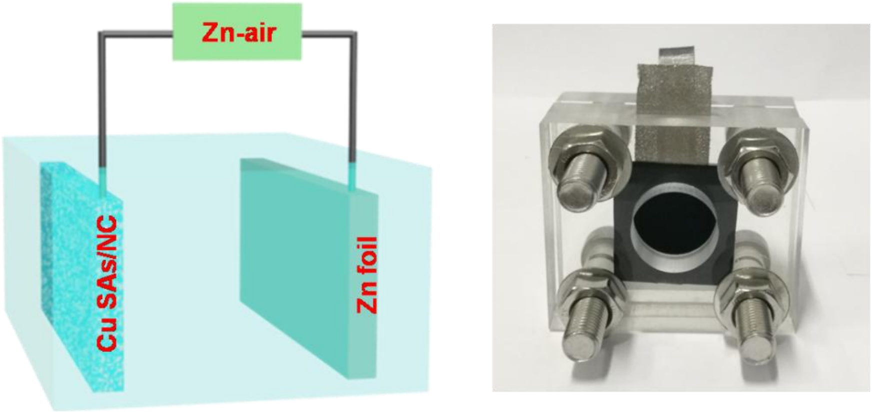

Supplementary Figure 20. Schematic illustration of the two-electrode primary Zn–air battery and a digital photograph of home-made Zn-air battery.

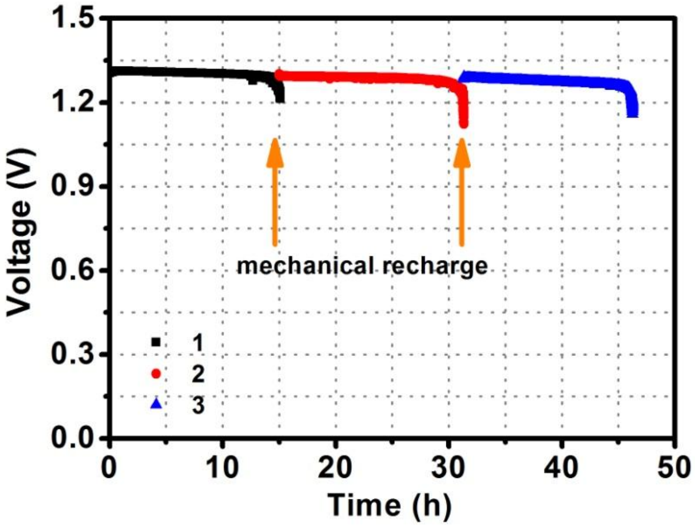

Supplementary Figure 21. Long-term stability of the primary Zn-air battery with Cu ISAS/NC cathode on a current density of 20 mA cm^{-2}. The battery was recharged by re-filling the Zn anode and electrolyte.

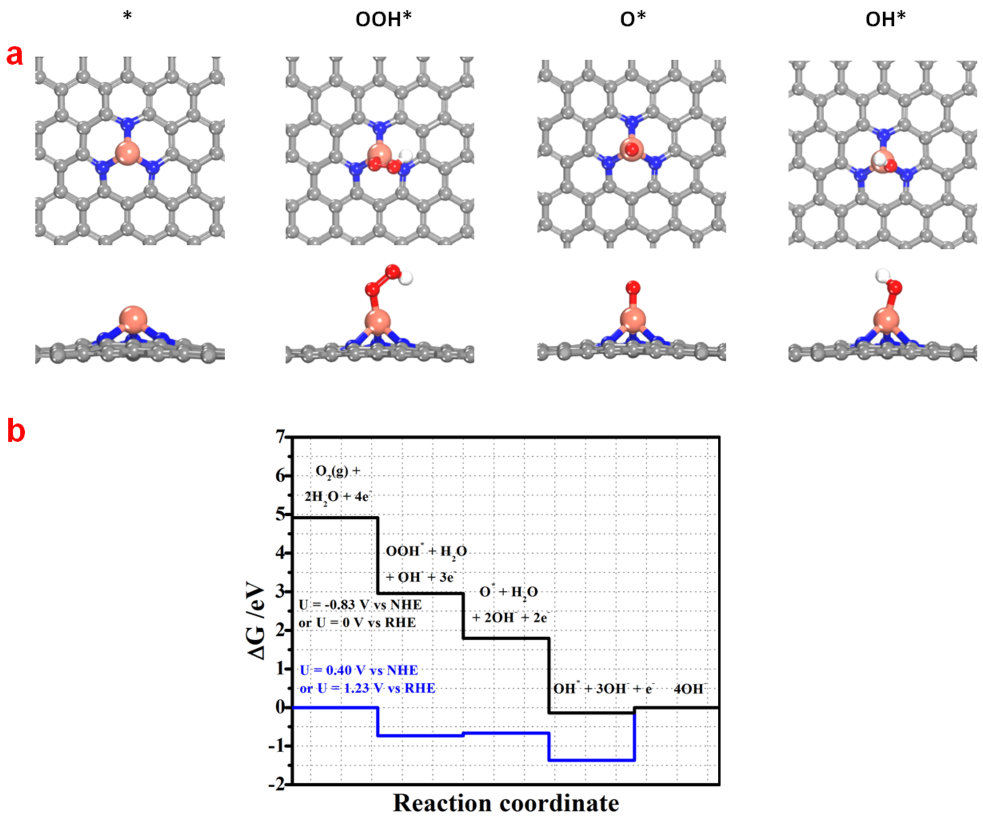

Supplementary Figure 22. a) Top and front views of the optimized structures of the models, adsorbed OOH, O and OH, respectively (from the left), over the Cu-N_3 model. Gray, blue, orange, red and white represent C, N, Cu, O and H atoms, respectively. b) Free energy diagram for ORR process on the Cu-N_3 model at the zero cell potential (U = -0.83 V vs NHE or U = 0 V vs RHE) and equilibrium potential (U = 0.40 V vs NHE or U = 1.23 V vs RHE) at pH = 14.

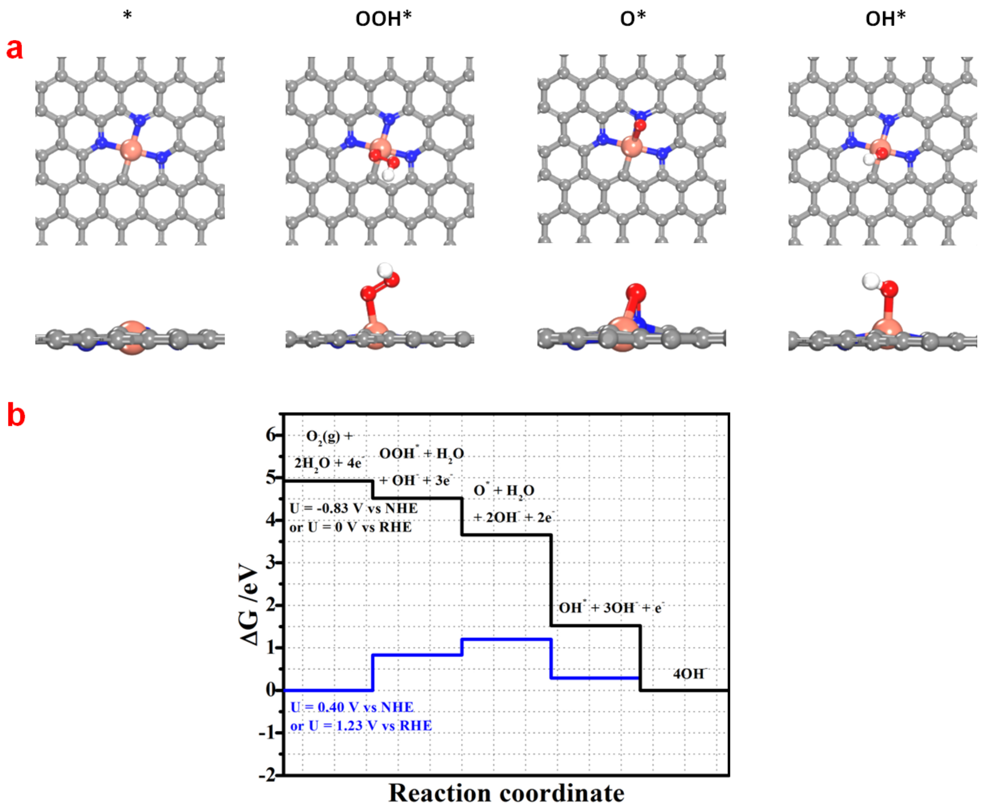

Supplementary Figure 23. a) Top and front views of the optimized structures of the models, adsorbed OOH, O and OH, respectively (from the left), over the Cu-N_3-C model. Gray, blue, orange, red and white represent C, N, Cu, O and H atoms, respectively. b) Free energy diagram for ORR process on the Cu-N_3-C model at the zero cell potential (U = -0.83 V vs NHE or U = 0 V vs RHE) and equilibrium potential (U = 0.40 V vs NHE or U = 1.23 V vs RHE) at pH = 14.

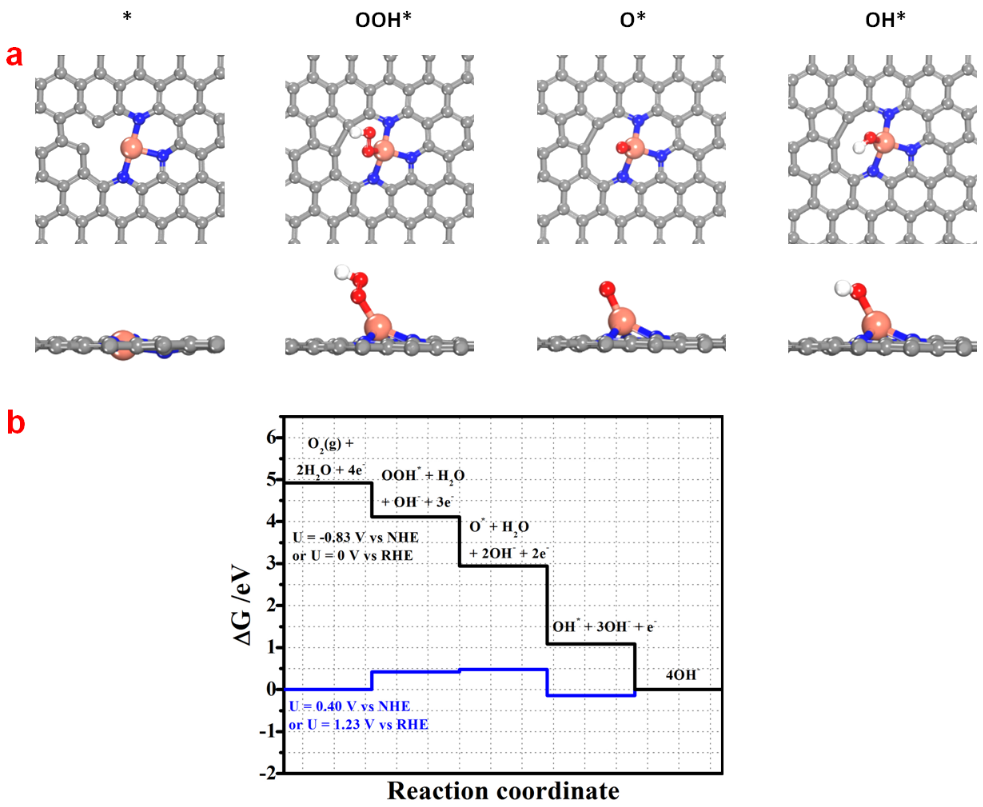

Supplementary Figure 24. a) Top and front views of the optimized structures of the models, adsorbed OOH, O and OH, respectively (from the left), over the Cu-N_3-V model. Gray, blue, orange, red and white represent C, N, Cu, O and H atoms, respectively. b) Free energy diagram for ORR process on the Cu-N_3-V model at the zero cell potential (U = -0.83 V vs NHE or U = 0 V vs RHE) and equilibrium potential (U = 0.40 V vs NHE or U = 1.23 V vs RHE) at pH = 14.

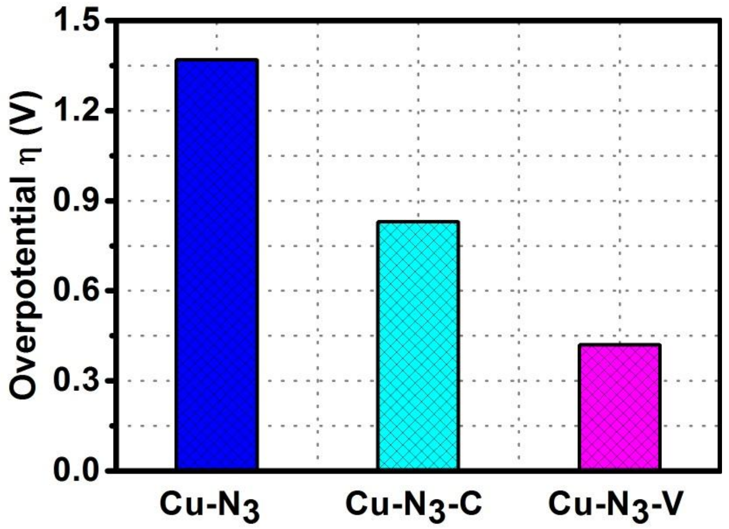

Supplementary Figure 25. The corresponding theoretical ORR overpotentials (η_{ORR}) for the three models.

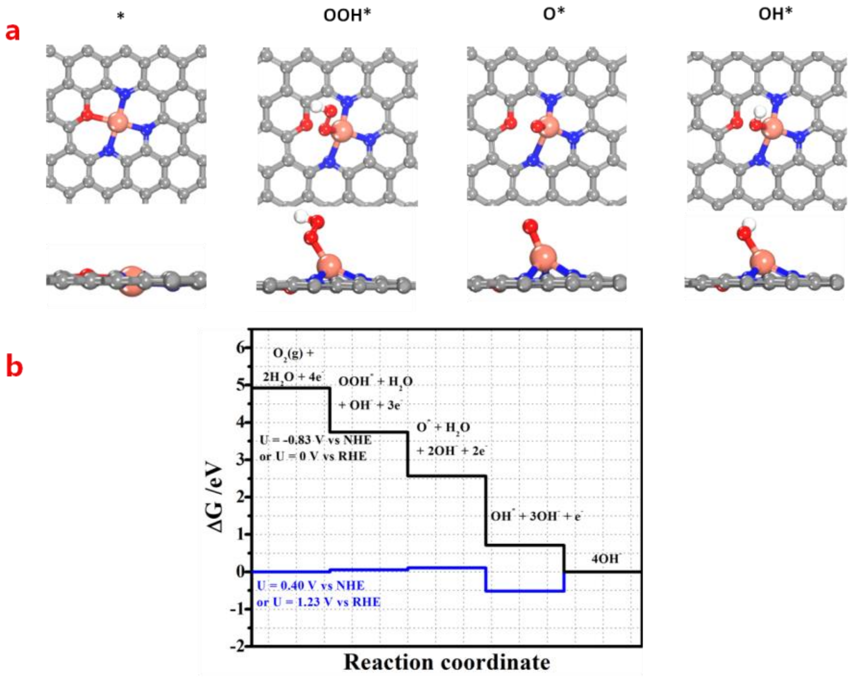

Supplementary Figure 26. Top and front views of the optimized structures of the model, adsorbed OOH, O and OH, respectively (from the left), over the O-preadsorbed Cu-N_3-V model (Cu-N_3-V-O-preadsorbed model). Gray, blue, orange, red and white represent C, N, Cu, O and H atoms, respectively. b) Free energy diagram for ORR process on the Cu-N_3-V-O-preadsorbed model at the zero cell potential (U = -0.83 V vs NHE or U = 0 V vs RHE) and equilibrium potential (U = 0.40 V vs NHE or U = 1.23 V vs RHE) at pH = 14.

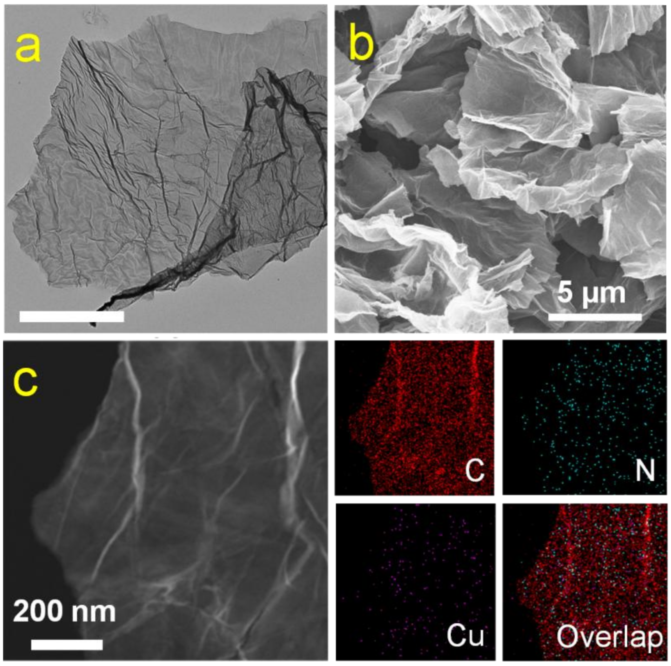

Supplementary Figure 27. The TEM a), SEM b) and EDS mapping c) of Cu ISAS/N-r-GO.

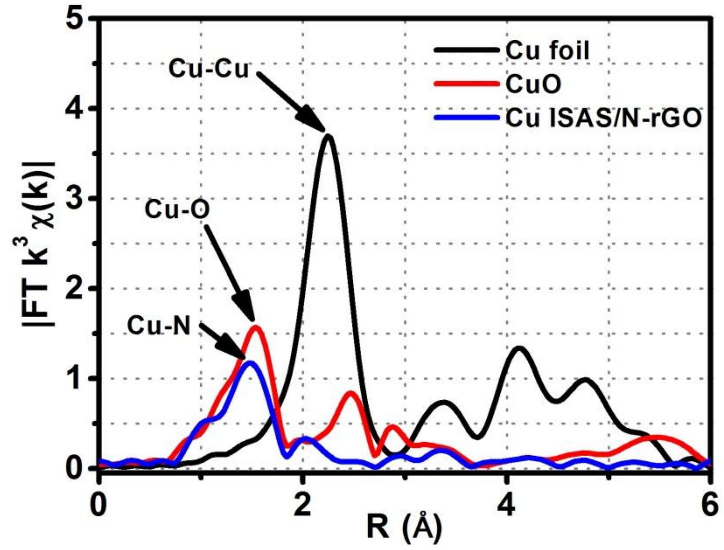

Supplementary Figure 28. FT k^3-weighted EXAFS spectra of Cu ISAS/N-rGO and the reference samples.

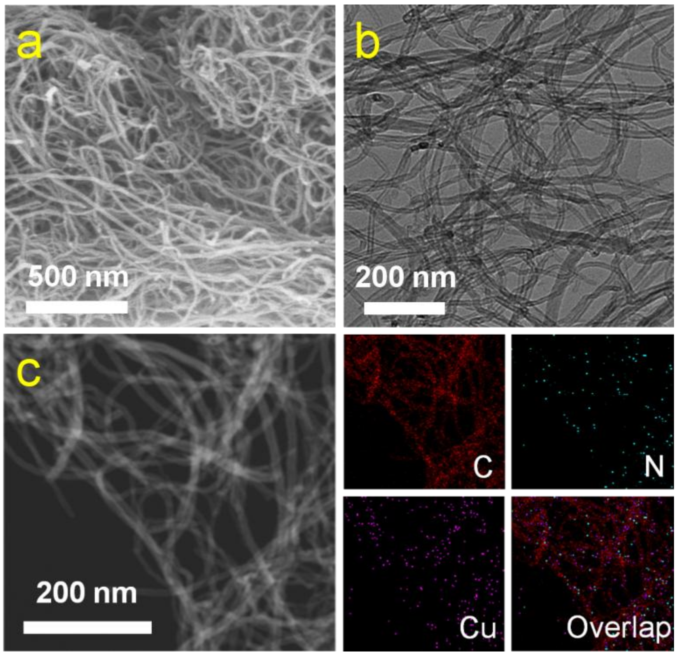

Supplementary Figure 29. The TEM a), SEM b) and EDS mapping (c) of Cu ISAS/N-CNTs.

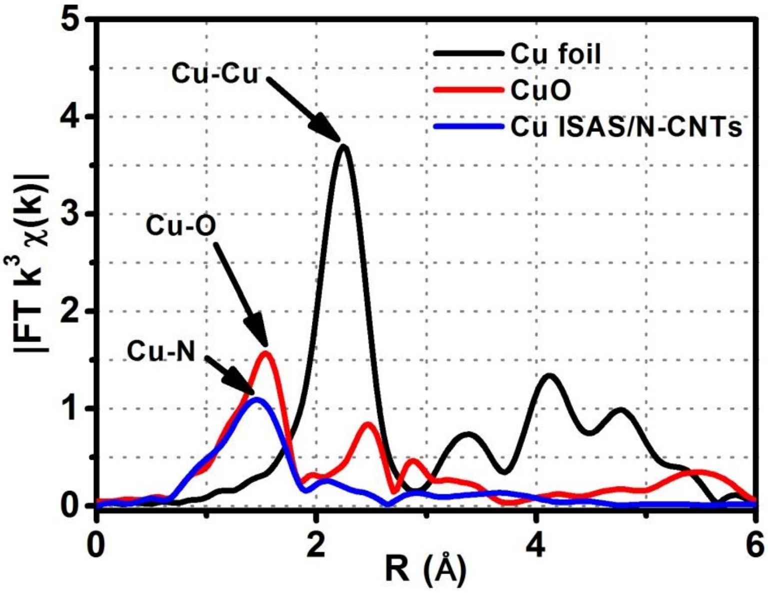

Supplementary Figure 30. FT k^3-weighted EXAFS spectra of Cu ISAS/N-CNTs and the reference samples.

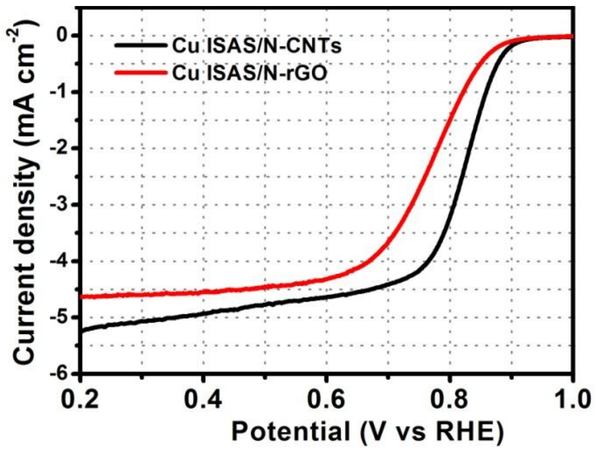

Supplementary Figure 31. LSV curves of Cu ISAS/N-CNTs and Cu ISAS/N-rGO catalysts in O_2-saturated 0.1 M KOH with sweep rate 10 mV s^{-1} and rotation rate 1600 rpm.

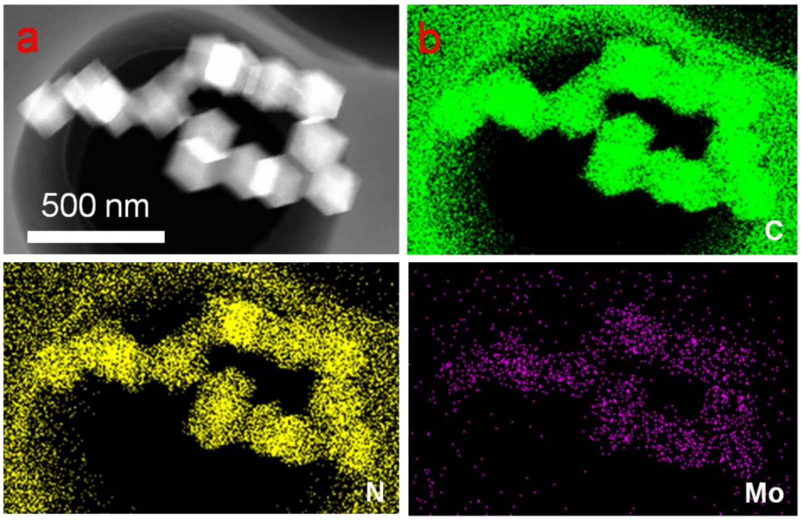

Supplementary Figure 32. a) The HAADF-STEM image and b) corresponding EDS mapping of Mo ISAS/NC.

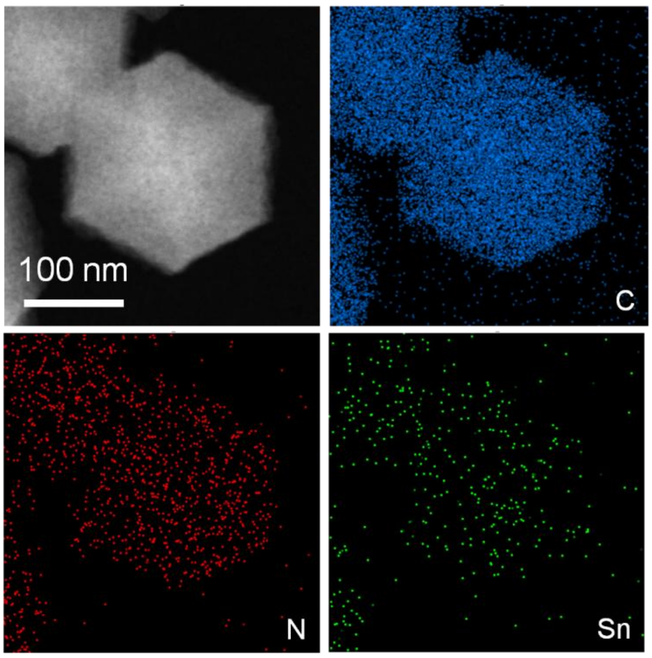

Supplementary Figure 33. The TEM image and corresponding EDS mapping of Sn ISAS/NC.

Supplementary Tables

Supplementary Table 1. Structural parameters extracted from the Cu K-edge EXAFS fitting. (S_0^2=0.85)

sample	Scattering pair	CN	R(Å)	$\sigma^2(10^{-3}Å^2)$	ΔE_0(eV)	R factor
Cu ISAS/NC	Cu-N	3.2	1.95	2.6	-2.3	0.0048

S_0^2 is the amplitude reduction factor; CN is the coordination number; R is interatomic distance (the bond length between central atoms and surrounding coordination atoms); σ^2 is Debye-Waller factor (a measure of thermal and static disorder in absorber-scatterer distances); ΔE_0 is edge-energy shift (the difference between the zero kinetic energy value of the sample and that of the theoretical model). R factor is used to value the goodness of the fitting.

Error bounds that characterize the structural parameters obtained by EXAFS spectroscopy were estimated as N ± 20%; R ± 1%; σ^2 ± 20%; ΔE_0 ±20%.

Supplementary Table 2. The Comparison of ORR performance of non-precious catalysts from the recent literature and this work (electrode 1600 rpm in 0.1 M KOH medium).

Catalysts	Half-wave potential	Onset potential (V vs RHE)	Electron transfer numbers	Reference
Cu ISAS/NC	0.92	1.05	3.99	**This work**
Cu-N-C	0.869	0.98	3.97	*Energy & Environ. Sci.* **2018**, 11, 2263.
LDH@ZIF-67−800	0.83	0.94	4.0	*Adv. Mater.* **2016**,28, 2337
Cu-N@C	~0.8	~0.92	3.96	*Energy Environ. Sci.* **2016**, *9*, 3736.
CoP-CMP800	0.81	0.85	3.94	*Adv. Mater.* **2014**, 26, 1450.
Fe@C-FeNC-2	0.899	1.04	4.0	*J. Am. Chem. Soc,* **2016**, *138*, 3570.
Fe-N-CNFs	0.85	0.93	3.95	*Angew. Chem. Int. Ed.* **2015**, 54, 8179.
FePhen@MOF-ArNH_3	0.86	1.03	4.0	*Nat. Commun.* **2015**, *6*, 7343.
Fe-ISAs/CN	0.9	0.99	3.9	*Angew. Chemie Int. Ed.* **2017**, 56, 6937.
Fe/NMC-11	0.862	1.0	3.9	*Adv. Energy Mater.* **2017**, 1701154.
CoII-A-rG-O	0.81	0.88	3.95	Angew. Chem. Int. Ed., 2015, 54, 12622.
NCNTFs	0.87	0.95	3.97	*Nature Energy* **2016**, 1, 15006.
CoOx NPs/BNG	0.805	0.95	4.0	*Angew. Chem. Int. Ed.* **2017**, *56*, 7121.
Fe/N/C HNSs.	0.77	0.9	3.80	*Nanoscale* **2015**, 7, 1501.
rGO/($Co2^{+}$–THPP)$_7$	/	0.86	3.85	*Angew. Chem. Int. Ed.* **2013**, 52, 5585.
Co–N–C–NS	0.84	0.93	3.7	*Nanoscale* **2015**, 7, 10334.
Fe/N/CNTAs	0.88	0.97	4.0	*Small* **2017**, 13, 1603407.
Fe−Nx−C	0.87	0.95	3.3	*Carbon*, **2014**, 78, 49.
Fe_3C@NG800−0.2	0.82	0.95	3.5	*ACS Appl. Mater. Interfaces* **2015**, 7, 21511.
Fe@C-FeNC-2	0.899	1.0	3.95	*J. Am. Chem. Soc.* **2016**,

				138, 3570.
Fe-N-SCCFs	0.883	1.03	3.9	*Nano Lett.* **2017**, *17*, 2003.
FeN4-GN	0.86	1.05	3.99	*Nano Energy* **2017**, *32*, 353.
CoO/NCNT	0.86	0.93	3.9	*J. Am. Chem. Soc.* **2012**, *134*, 15849.
Co-N-C	0.871	0.98	3.4-4.0	*ACS Catal.* **2015**, 5, 7068.
P-CNCo-20	0.85	0.93	3.9	*Adv. Mater.* **2015**, *27*, 5010.

Supplementary Table 3. Comparison of peak power density of different primary Zinc-air batteries reported in literatures.

**Catalyst**	**Catalyst loading (mgcm^{-2})**	**Peak power density (mW cm^{-2})**	**Reference**
Cu ISAS/NC	1	280	**This work**
CuPt-NC	2	251	*ACS Catal.* **2015**, *5*, 1445.
Fe@N–C-700	2.2	220	*Nano Energy* **2015**, *13*, 387.
Mn_3O_4/graphene	2	120	*Energy Environ. Sci.* **2011**, *4*, 4148.
CoO/N-CNT	1	265	*Nat. Commun.* **2013**, *4*, 1805.
N-CNT	0.2	70	*Electrochim. Acta* **2011**, *56* , 5080.
Co_4N/CNW/CC	1	174	*J. Am. Chem. Soc.* **2016**, *138*, 10226.
FeCo-N-C	1.5	232	*J. Power Sources* **2011**, *196, 3673.*
Co_3O_4/MnO_2	2	33	*Nanoscale* **2013**, *5*, 4657.
Fe-N-MC	1.2	190	*Nano Energy* **2016**, *26*, 131.
Co-doped TiO_2	2	136	*J. Mater. Chem. A* **2016**, *4*, 7841.
Co_3O_4–SP/NGr	1	190	*ACS Appl. Mater. Interfaces* **2015**, *7*, 21138.
Cu-N@C	0.4	210	*Energy Environ. Sci.* **2016**, *9*, 3736.

Supplementary Table 4. Reaction free energy of elementary step for ORR at U_{NHE} = 0.40 V at pH = 14 as well as the theoretical overpotential ($U_{overpoetntial}$, eV) on the Cu-N_3, Cu-N_3-C and Cu-N_3-V.

Samples	U_{NHE} = 0.40 V				$U_{overpoetntial}$
	ΔG_1	ΔG_2	ΔG_3	ΔG_4	
Cu-N_3	-0.73	0.07	-0.71	1.37	1.37
Cu-N_3-C	0.83	0.37	-0.91	-0.29	0.83
Cu-N_3-V	0.42	0.06	-0.63	0.15	0.42
