# Supplementary material for: Directly transforming copper (I) oxide bulk into isolated single-atom copper sites catalyst through gas-transport approach
Source: Nat Commun. 2019 Aug 19;10:3734. doi: 10.1038/s41467-019-11796-4 (PMC6700197; doi:10.1038/s41467-019-11796-4)
Supplement: Supplementary file 2 — Description of Additional Supplementary Files [file 41467_2019_11796_MOESM2_ESM.docx]

**Description of Additional Supplementary Files**

File Name: Supplementary Data 1
Description: Optimized structures of Cu-N3 model zip

File Name: Supplementary Data 2
Description: Optimized structures of Cu-N3-C model

File Name: Supplementary Data 3
Description: Optimized structures of Cu-N3-V model

File Name: Supplementary Data 4
Description: Optimized structures of Cu-N3-V-O-preadsorbed model
